# External validation of FRISBEE 2-year and 5-year fracture prediction models in a fracture liaison service cohort

**DOI:** 10.1007/s11657-025-01516-5

**Published:** 2025-08-02

**Authors:** Tove T. Borgen, Cathrine Brunborg, Frede Frihagen, Lene B. Solberg, Camilla Andreasen, Wender Figved, Ellen M. Apalset, Jan-Erik Gjertsen, Trude Basso, Jens-Meinhard Stutzer, Lars Nordsletten, Erik F. Eriksen, Åshild Bjørnerem

**Affiliations:** 1 Department of Rheumatology, Vestre Viken Hospital Trust, Drammen Hospital, Postbox 800, N-3004 Drammen, Norway; 2 Oslo Centre for Biostatistics and Epidemiology, Research Support Services, Oslo University Hospital, Oslo, Norway; 3 Department of Orthopedic Surgery, Østfold Hospital, Kalnes, Norway; 4 Institute of Clinical Medicine, University of Oslo, Oslo, Norway; 5 Division of Orthopedic Surgery, Oslo University Hospital, Oslo, Norway; 6 Department of Clinical Medicine, Uit, the Arctic University of Norway, Tromsø, Norway; 7 Department of Orthopedic Surgery, University Hospital of North Norway, Tromsø, Norway; 8 Department of Orthopedic Surgery, Vestre Viken Hospital Trust, Bærum Hospital, Gjettum, Norway; 9 Bergen Group of Epidemiology and Biomarkers in Rheumatic Disease, Department of Rheumatology, Haukeland University Hospital, Bergen, Norway; 10 Department of Global Public Health and Primary Care, University of Bergen, Bergen, Norway; 11 Department of Orthopedic Surgery, Haukeland University Hospital, Bergen, Norway; 12 Department of Clinical Medicine, University of Bergen, Bergen, Norway; 13 Department of Orthopedic Surgery, St. Olavs University Hospital, Trondheim, Norway; 14 Department of Orthopedic Surgery, Møre and Romsdal Hospital Trust, Molde Hospital, Molde, Norway; 15 Faculty of Dentistry, University of Oslo, Oslo, Norway; 16 Pilestredet Park Specialist Centre, Oslo, Norway; 17 Norwegian Research Centre for Women’s Health, Oslo University Hospital, Oslo, Norway; 18 Department of Obstetrics and Gynecology, University Hospital of North Norway, Tromsø, Norway

**Keywords:** DXA, Fracture risk assessment, Osteoporosis

## Abstract

**Summary:**

We externally validated the FRISBEE models of 2-year and 5-year fracture risk prediction in 517 women with index fractures. Both models overestimated the fracture risk. Recalibration of the FRISBEE models are needed before use in Norwegian women with recent fractures.

**Purpose:**

We externally validated the Fracture Risk Brussels Epidemiological Enquiry (FRISBEE) groups’ 2-year and 5-year fracture risk models.

**Methods:**

We included women above 50 years with a recent fracture from the consent-based part of the Norwegian Capture the Fracture Initiative study (NoFRACT). They had bone mineral density assessed and filled in a questionnaire including risk factors for fracture at baseline between October 2015 and December 2017. We calculated and validated the 2-year and 5-year fracture risk using the FRISBEE equation models.

**Results:**

Of 517 women aged 65.5 ± 8.6 years with fractures, 94 (18%), 55 (11%), and 31 (6%) sustained a subsequent fracture of any type, major osteoporotic fractures (MOF), or central fracture, during 4.7 ± 1.3 years mean follow-up. The area under the receiver-operating curve (AUC) (95% confidence interval (CI)) for any type of fracture, MOF, and central fracture was 0.57 (0.51–0.63), 0.57 (0.46–0.67), and 0.65 (0.53–0.77), respectively, for the FRISBEE 2-year risk models and 0.57 (0.51–0.64), 0.58 (0.50–0.67), and 0.67 (0.57–0.76) for the FRISBEE 5-year risk models. The calibration slopes (with 95% CI) that compared observed vs. predicted probabilities for fracture across deciles of risk for any type of fracture, MOF, and central fracture were all low: 0.34 (0.02–0.64), 0.33 (− 0.09–0.74), and 0.61 (0.16–1.06), in the FRISBEE 2-year models, and 0.54 (0.13–0.95), 0.43 (0.05–0.80), and 0.69 (0.31–1.08), in the FRISBEE 5-year models.

**Conclusion:**

Overall, the FRISBEE models overestimated both 2-year and 5-year fracture risk. Recalibration is needed before these models can be used in Norwegian women with recent fractures.

**Supplementary Information:**

The online version contains supplementary material available at 10.1007/s11657-025-01516-5.

## Introduction

Calculation of an individual’s absolute fracture risk to identify patients eligible for treatment with anti-osteoporotic drugs (AOD) is recommended in several guidelines [1, 2]. Clinical risk factors in addition to bone mineral density (BMD) and prior vertebral or non-vertebral fractures enhance the accuracy of prediction of hip and major osteoporotic fractures (MOF) in women and men [3]. The most used fracture risk prediction tools are Garvan nomograms [4], where 5-year and 10-year fracture risk can be estimated, and the Fracture Risk Assessment Tool (FRAX) [5], where 10-year fracture risk can be estimated.

The Fracture Risk Brussels Epidemiological Enquiry (FRISBEE) group in Belgium has also developed 5-year risk models for fractures from the prospective FRISBEE cohort [6]. These models predict risk of any type of fracture (like the Garvan nomograms), MOF (like FRAX), and central fracture (fracture of the hip, spine, shoulder, pelvis, ribs, or clavicle).

The fracture risk is elevated during the first 2 years following a fracture, known as the “imminent fracture risk” [7, 8]. Awareness and estimation of imminent fracture risk is important in secondary fracture prevention. Garvan and the conventional FRAX models have not been modified to calculate imminent fracture risk, but this is provided as one of the FRAXplus options [9]. The FRISBEE group has recently also developed models for predicting imminent fracture risk [10]. In these models, only a few of the clinical risk factors (CRF) from the Garvan and FRAX models are included. Three different models have been constructed to estimate the 2-year risk of (i) any type of fracture, (ii) MOF, and (iii) central fracture [11]. They have also taken into account that some studies have indicated that the risk of a subsequent fracture is higher following certain types of fractures, especially central fractures [6, 11]. As a result, the FRISBEE group has included an incident central fracture as a risk factor for prediction of both imminent MOF and central fracture.

The FRISBEE 5-year risk models for fracture have been externally validated in a Canadian population [12]. In these validations, the fracture risk was overestimated (almost doubled). The authors concluded that recalibration was necessary to fit the Canadian population. It was therefore of interest to validate these models in other cohorts. Furthermore, the FRISBEE 2-year models to estimate imminent fracture risk have so far not been externally validated. We were asked by the FRISBEE group to validate these models and to study their ability to predict the 2-year and 5-year risk of fractures in a cohort of patients with fractures from the Norwegian Capture the Fracture Initiative (NoFRACT) trial [13]. It was also of interest to compare the results with the validation of the Garvan nomogram and FRAX in the same cohort.

## Material and methods

### Study subjects

NoFRACT (NCT02536898) was conducted at the orthopedic departments at seven hospitals in Norway from 6 May 2015 to 31 December 2018 with 34,976 patients enrolled [13]. The objectives of NoFRACT were to improve secondary fracture prevention by introducing a fracture liaison service (FLS) model of care for identification, assessment, and treatment of osteoporosis in patients who recently had sustained an index fracture, and to investigate the effect of the intervention on the rate of subsequent fractures and mortality. All women and men above 50 years with fractures were eligible for intervention, except those with fractures in toes, fingers, skull, and face.

**Fig. 1:**
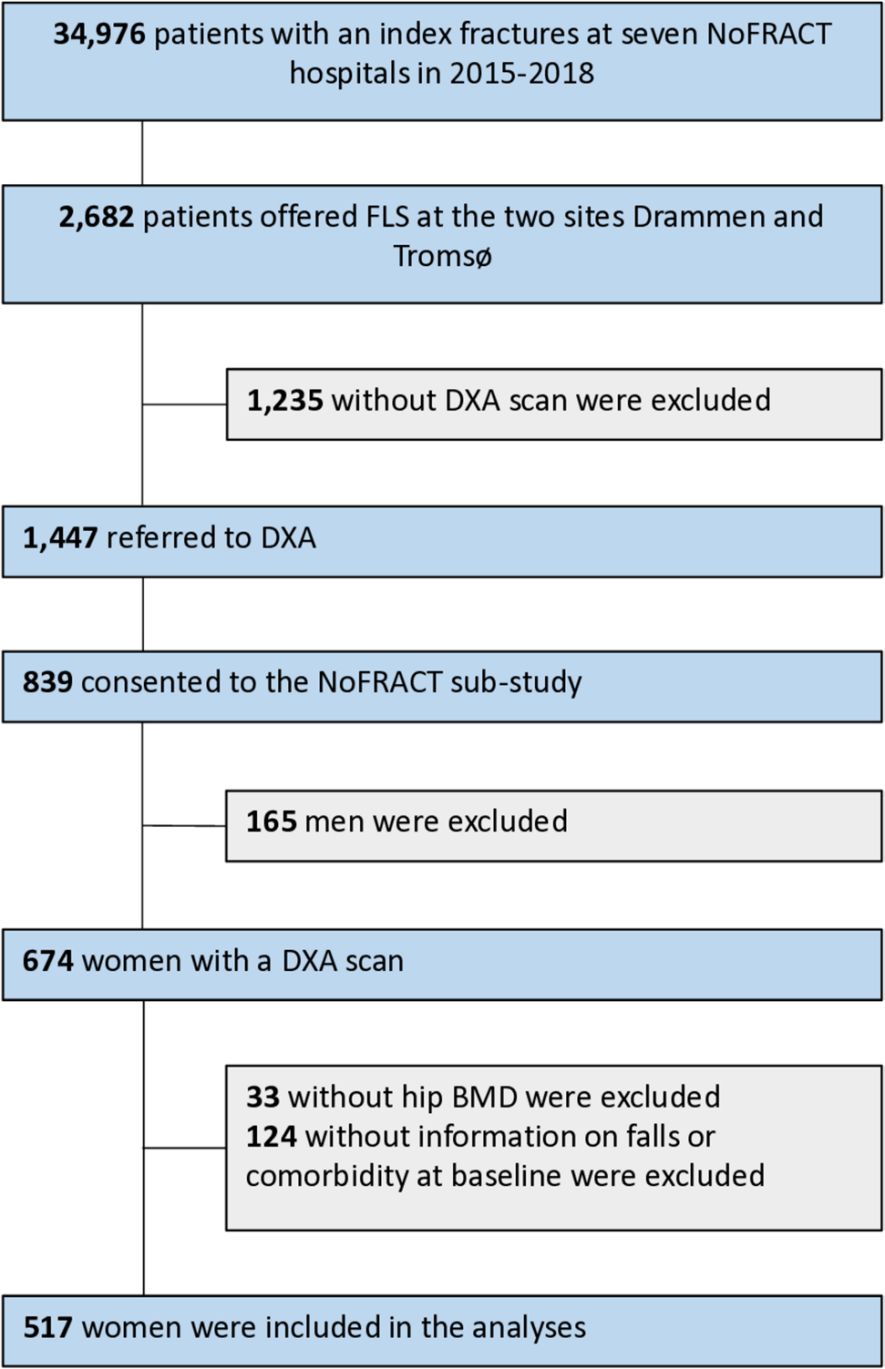
Flow-chart of the study participants. NoFRACT, Norwegian Capture the Fracture Initiative study; DXA, dual energy X-ray absorptiometry; FRAX, Fracture Risk Assessment Tool; BMD, bone mineral density

A sub-study of NoFRACT (NCT02608801) was conducted at two of the seven participating hospitals [14]. Of all 2682 recruited patients with a recent fracture at Drammen Hospital from 1 January 2016 to 31 December 2017 (*n* = 1838) and the University Hospital of North Norway, Tromsø from 1 October 2015 to 31 December 2017 (*n* = 844), 1447 were referred to dual energy x-ray absorptiometry (DXA) (Fig. 1). Of these, 839 patients provided written, informed consent to participate in the NoFRACT sub-study. We excluded 165 men. Of 674 women, 33 were excluded due to missing BMD measurement of at least one hip, and 124 were excluded due to insufficient information on falls and/or comorbidity at the baseline questionnaire. Hence, 517 women were included in the analyses of the present study using the FRISBEE models. The NoFRACT sub-study and this validation study of the FRISBEE models were approved by the Regional Committee for Medical and Health Research Ethics (REK 2014/2260) and was conducted in accordance with the World Medical Association Declaration of Helsinki.

### Variables

The index fracture (the fracture that led to inclusion in NoFRACT) and the first fracture occurring during follow-up until October 2021 were categorized according to the location of the fracture: (1) any type of fracture (all types of fracture except craniofacial, hand, and foot), (2) MOF (vertebra, hip, proximal humerus, and forearm), and central fracture (vertebrae, hip, proximal humerus, clavicula, ribs, and pelvis). The localization, number, and time of injuries with fracture during follow-up were registered carefully from the patient records to ensure that the fractures were not double-counted, and only data of the first new incident fracture was used in the analyses.

Height and weight were measured at baseline and body mass index (BMI) was calculated. BMD was measured using GE Lunar iDXA (GE Healthcare, Madison, WI, USA) in Drammen and GE Lunar Prodigy Pro (GE Healthcare, Madison, WI, USA) in Tromsø. Quality assurance with daily phantom test of the DXA equipment was performed as recommended. BMD was measured at the lumbar spine, femoral neck, and total hip, and BMD of the left hip was used, except in 32 women who did not have measurable left hip, in which case the measurement of the right hip was used instead. In the FRISBEE study, hologic equipment was used for the evaluation of BMD; hence, the equations proposed by Fan et al. were used to convert BMD measurements by GE Lunar to hologic BMD at lumbar spine, femoral neck, and total hip [15]. For each patient, we calculated the 10-year risk of any type of fracture using the online Bone Fracture Risk Calculator of the Garvan Institute of Medical Research (www.garvan.org.au) and the 10-year risk of MOF using the online FRAX calculator (www.FRAX.shef).

Information on falls, hip fractures in parents, comorbidities, rheumatoid arthritis, the use of glucocorticoids, smoking, and alcohol habits were recorded from a baseline questionnaire. Patients were registered with “a history of fall” if they had one or more falls during the year preceding baseline, and the fall that lead to the index fracture was not counted. We recorded adherence to AOD at 2-year follow-up. As most patients who are not adherent to prescribed medication stop taking the drug during the first year, we assumed that the patients who still were adherent to their medication after 2 years were most likely adherent after that. Fractures and deaths were registered from patient records after mean follow-up time of 4.7 years (range 0–5.6 years).

The 2-year and 5-year probability of any type of fracture, MOF, and central fracture were calculated for each subject using the FRISBEE equations provided by the inventors, in Excel sheets with the generated formulas, including significant risk factors for each model. The risk factors included in the 2-year model were for any type of fracture (total hip BMD, history of falls, and comorbidities), for MOF (femoral neck BMD, age, history of falls, comorbidities, and central index fracture), and for central fracture (femoral neck BMD, age, history of falls, comorbidities, and central index fracture) [11]. The risk factors included in the 5-year model were as follows: for any type of fracture (total hip BMD, age, history of fragility fracture, and history of falls), for MOF (total hip BMD, lumbar spine BMD, age, history of fragility fracture, and excessive alcohol intake (≥ 3 units/day)), and for central fractures (total hip BMD, lumbar spine BMD, age, history of fragility fracture, and rheumatoid arthritis) [6].

### Statistical analyses

Continuous variables were reported as mean ± standard deviation (SD). Information on comorbidities (including rheumatoid arthritis), use of glucocorticoids and AOD, smoking, alcohol intake ≥ 3 units per day, falls, and subsequent fractures were dichotomized and reported as number (%).

The FRISBEE models predict 2-year [10] and 5-year [6] fracture risk by the Fine and Gray competing risk model [16], taking into account the competing risk of death and differences in follow-up time, since this was a dynamic cohort study. Follow-up time in patient-years was calculated from the date of the index fracture until the date of the first incident fracture (during follow-up), death or end of follow-up (October 2021), whichever occurred first. The model’s formulas and coefficients for the FRISBEE scores were obtained from the FRISBEE group’s original paper [10]. We validated the scores by discrimination and calibration using competing risk models. Discrimination, which measures the risk scores’ ability to differentiate between those who experienced a fracture during the timespan of the model versus those who did not, was evaluated by area under the receiver-operating characteristic (ROC) curves (AUC). If the AUC is > 0.7, it can be concluded that the model has an acceptable discriminatory capability [17].

Calibration was evaluated by comparing observed versus predicted probabilities of incident fractures. This was done by comparing probabilities across deciles of risk and graphically by calibration plots. In a plot of observed vs predicted probabilities, perfect calibration will be on the 45 $^\circ$ line. In addition, we calculated the calibration slope which is a measure of agreement between observed and predicted risk of the outcome across the whole range of predicted values. It should ideally attain a value of 1.

The FRISBEE models were intended for individuals aged 60–85 years. We applied the models on all patients aged 50–90 years to achieve the highest possible number of participants, but also in sensitivity analyses in those aged 60–85 years.

We also explored the performance of the Garvan risk score for any type of fracture, MOF, and central fracture. We used the Garvan equation formula [18] for women to estimate 5-year risk of fracture for each individual patient in our cohort. The performance of FRAX in our cohort was also explored. As the FRAX formula is not open, we used the calculated 10-year fracture risk and multiplied by a factor of 0.47 to adjust for the mean follow-up time of 4.7 years [19]. The performance of the risk scores was evaluated by calibration and discrimination as described above. Cox’s proportional hazards model was used to evaluate the Garvan and FRAX risk scores. No sample size estimation was performed, since we were asked to validate the FRISBEE models using the data that was collected from the sub-study of NoFRACT. The statistical analyses were performed using Stata (Version 18, StataCorp LP, TX, USA).

## Results

All 517 women, with a mean age of 65.5 years (SD 8.6), had recently sustained an index fracture before inclusion in the study (Table 1). Of those, 339 (65.6%) had sustained a MOF, and 142 (27.5%) had sustained a central fracture. A total of 122 (23.6%) women reported one or more falls the last year before inclusion. At least one comorbidity was registered in 122 (23.6%) women, and 242 (56.1%) were taking AOD at 2-year follow-up. A total of 26 patients (5%) died during the mean follow-up time of mean 4.7 years (range 0–5.6).

**Table 1:** Characteristics of all the 517 participating women with index fractures

Index fracture types:	
MOF, *n* (%)	339 (65.6)
Central fracture, *n* (%)	142 (27.5)
Hip, *n* (%)	33 (6.4)
Distal forearm, *n* (%)	204 (39.5)
Proximal humerus, *n* (%)	74 (14.3)
Spine, *n* (%)	28 (5.4)
Ankle, *n* (%)	90 (17.4)
Pelvis, *n* (%)	7 (1.4)
Other, *n* (%)	81 (15.7)
Baseline characteristics	
Age (years), mean ± SD	65.5 ± 8.6
Body mass index kg/m^2^, mean ± SD	26.6 ± 4.6
Patients with falls the last year before inclusion, *n* (%)	122 (23.6)
Parents with hip fracture, *n* (%), of 481 observations	101 (21.0)
Current smoker, *n* (%), of 501 observations	73 (14.5)
Alcohol consumption ≥ 3 units per day, *n* (%)	5 (1.0)
Use of glucocorticoids, *n* (%), of 507 observations	26 (5.1)
Rheumatoid arthritis, *n* (%)	22 (4.3)
Comorbidities, *n* (%)	122 (23.6)
BMD of lumbar spine, g/cm^2^, mean ± SD	0.855 ± 0.178
BMD of total hip, g/cm^2^, mean ± SD	0.770 ± 0.114
BMD of femoral neck, g/cm^2^, mean ± SD	0.638 ± 0.097
Estimated risk scores	
FRISBEE 2-year risk score (%), for any type of fracture	25.4 ± 12.9
FRISBEE 2-year risk score (%), for MOF	6.5 ± 6.4
FRISBEE 2-year risk score (%), for central fracture	9.9 ± 9.5
FRISBEE 5-year risk (%), for any type of fracture	16.1 ± 7.6
FRISBEE 5-year risk (%), for MOF	7.2 ± 3.4
FRISBEE 5-year risk (%), for central fracture	11.2 ± 9.4
FRAX 10-year risk (%), for MOF	20.1 ± 10.1
Garvan 10-year risk of any type of fracture	40.3 ± 22.1
Using AOD treatment after the FLS-intervention, *n* (%)	242 (56.1)
Follow-up time, year, mean ± SD (range)	4.7 ± 1.3 (0–5.6)
Fractures during follow-up	
0–2 years any type of fracture, *n* (%)	53 (10.3)
0–2 years MOF, *n* (%)	35 (6.8)
0–2 years central fracture, *n* (%)	22 (4.3)
0–5.6 years any type of fracture, *n* (%)	94 (18.3)
0–5.6 years MOF, *n* (%)	55 (10.6)
0–5.6 years central fracture, *n* (%)	31 (6.0)

Values are mean ± SD or *n* (percent). *BMD*, bone mineral density; *MOF*, major osteoporotic fracture; *AOD*, anti-osteoporotic drugs; *Garvan*, Garvan nomograms; *FRAX*, Fracture Risk Assessment Tool; *FLS* fracture liaison service

The mean FRISBEE 2-year risk score for any type of fracture, MOF, and central fracture were 25.4%, 6.5%, and 9.9%, respectively (Table 1). The mean FRISBEE 5-year risk score for any type of fracture, MOF, and central fracture was 16.1%, 7.2%, and 11.2%, respectively.

During the first 2 years of follow-up, 53 (10.3%) women sustained a subsequent fracture of any type, 35 (6.8%) a MOF, and 22 (4.3%) a central fracture (Table 1). These numbers were accurate for MOF, but less than half of the expected numbers for any type of fractures and central fractures, using the FRISBEE 2-year prediction model.

During the mean follow-up of 4.7 years, 94 (18.3%) sustained a subsequent fracture of any type, 55 (10.6%) a MOF, and 31 (6.0%) a central fracture. These numbers were almost the same as expected according to the FRISBEE 5-year models for any type of fractures and MOF, but about half of what was expected for central fractures (Table 1).

### Discriminative performance of the models

**Fig. 2:**
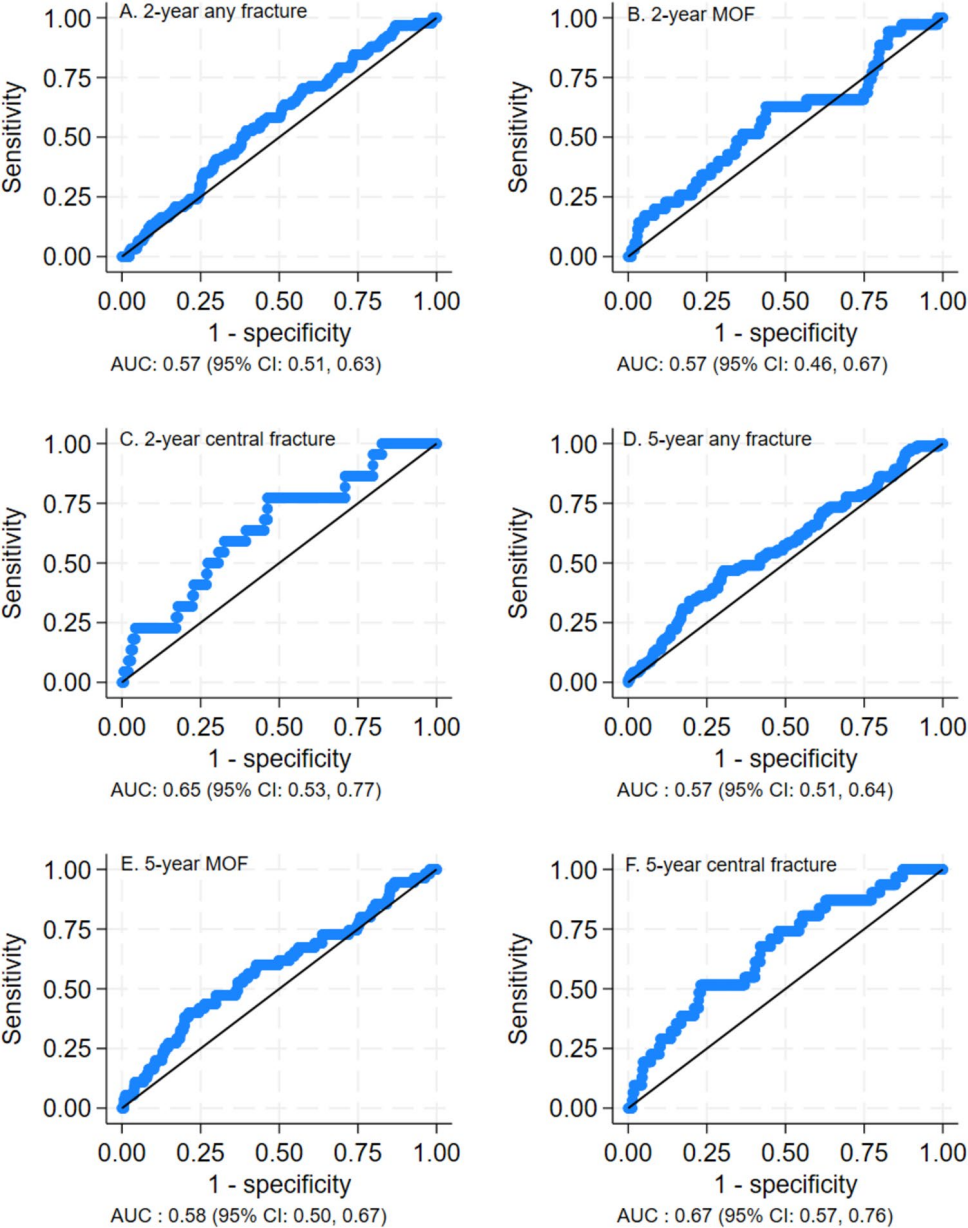
Area under the receiver-operating characteristic (ROC) curves (AUC) showing the FRISBEE 2-year scores’ ability to discriminate between those who experienced a fracture versus those who did not, for any type of fractures (**A**), major osteoporotic fractures (MOF) (**B**), and central fractures (**C**), and the FRISBEE 5-year risk scores’ ability to discriminate between those who experienced a fracture versus those who did not, for any type of fractures (**D**), major osteoporotic fractures (MOF) (**E**), and central fractures (**F**)

In the FRISBEE 2-year models, AUC statistics for discriminating fracture of any type, MOF, or central fracture were 0.57, 0.57, and 0.65, respectively (Table 2, Fig. 2A–C). In the FRISBEE 5-year models, AUC statistics for discriminating fracture of any type, MOF, or central fracture were 0.57, 0.58, and 0.67, respectively (Table 2, Fig. 2D–F). Sensitivity analyses including individuals aged 60–85 years did not improve the discriminative performance.

**Table 2:** Discriminative performance for 2-year and 5-year FRISBEE models, 5-year Garvan and 5-year FRAX for any type of fractures, major osteoporotic fractures and central fractures in the total cohort, and those aged 60–85 years, expressed in area under the receiver-operating characteristic curve with 95% confidence intervals

	Fracture	Total cohort	Aged 60–85 years
FRISBEE 2-year	Any type	0.57 (0.51–0.63)	0.55 (0.48–0.63)
	Major osteoporotic	0.57 (0.46–0.67)	0.56 (0.44–0.68)
	Central	0.65 (0.53–0.77)	0.60 (0.47–0.73)
FRISBEE 5-year	Any type	0.57 (0.51–0.64)	0.56 (0.49–0.64)
	Major osteoporotic	0.58 (0.50–0.67)	0.57 (0.47–0.66)
	Central	0.67 (0.57–0.76)	0.59 (0.47–0.71)
Garvan 5-year	Any type	0.59 (0.53–0.66)	0.61 (0.53–0.68)
	Major osteoporotic	0.61 (0.53–0.70)	0.63 (0.54–0.72)
	Central	0.70 (0.60–0.80)	0.66 (0.55–0.76)
FRAX* 5-year	Major osteoporotic	0.62 (0.54–0.70)	0.63 (0.55–0.72)

*AOD*, anti-osteoporotic drugs; *Garvan*, Garvan nomograms; *FRAX*, Fracture Risk Assessment Tool. *Missing FRAX calculation for 7 patients, *n* = 510

In comparison, the discriminative performance of Garvan 5-year risk models for any type of fracture, MOF, and central fracture revealed an AUC of 0.59, 0.61, and 0.70, respectively (Table 2, Fig. S1). The discriminative performance of FRAX for MOF showed an AUC of 0.62 (Table 2, Fig. S1).

### Calibration of the models

**Fig. 3:**
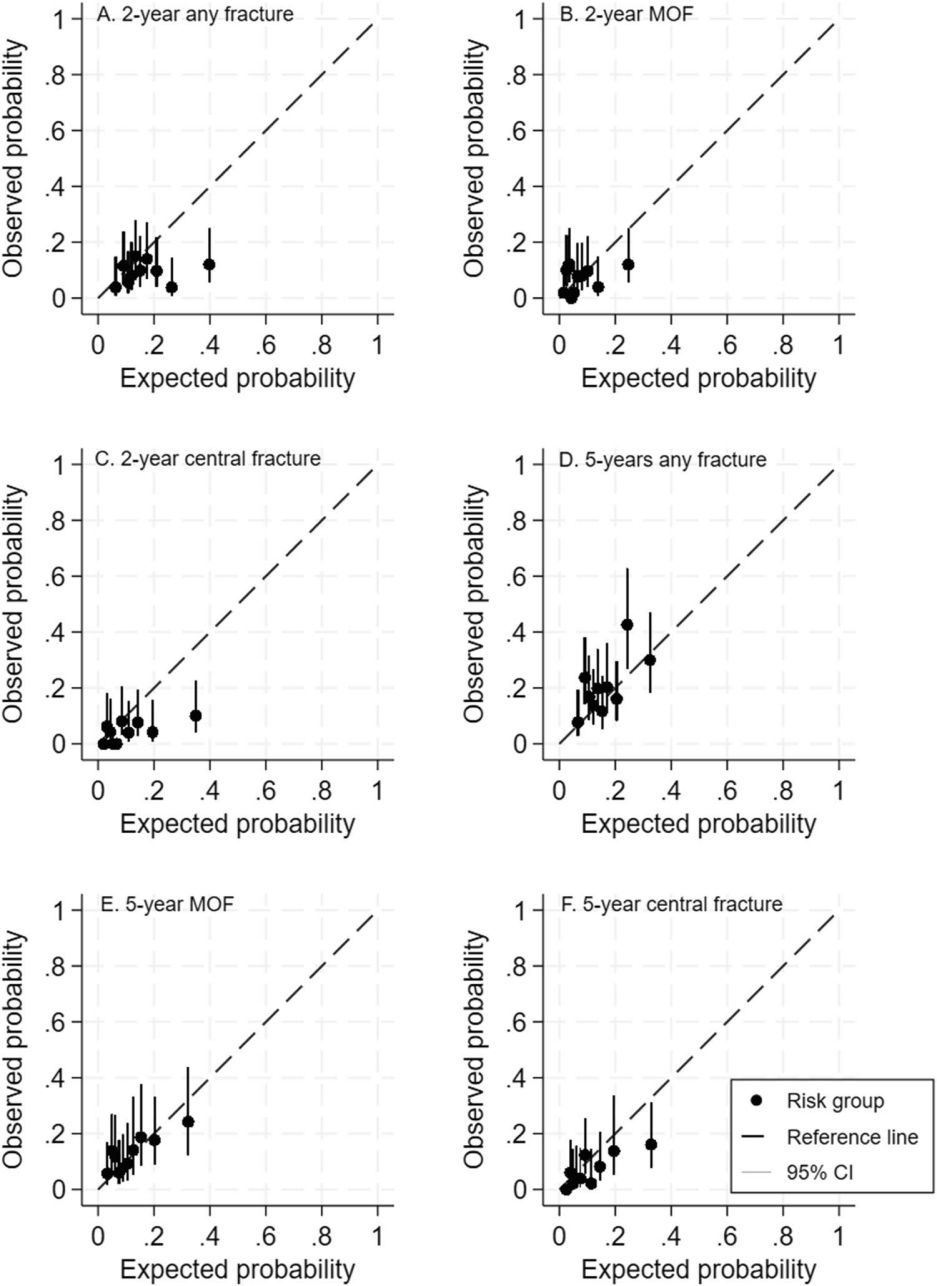
Calibration plots of the FRISBEE 2-year fracture risk prediction models across deciles of risk for any type of fractures (**A**), major osteoporotic fractures (**B**), and central fractures (**C**) and calibration curves of the FRISBEE 5-year fracture risk prediction models for any type of fractures (**D**), major osteoporotic fractures (**E**), and central fractures (**F**)

In the FRISBEE 2-year risk models, calibration plots showed poor agreement between observed and predicted probability across the deciles of risks for all three types of fractures, and all the deciles of risk fell below the unity line in the calibration plot (Fig. 3A–C). Most of the deciles were clustered at the bottom left. The calculated calibration slopes were low for any fractures (0.34), MOF (0.33), and moderate for central fractures (0.61) (Table 3).

**Table 3:** Calibration slopes (95% confidence interval) of agreement between observed and predicted risk of fractures for the FRISBEE 2-year and 5-year risk models and for the 5-year Garvan and FRAX models

	Fracture	Total cohort	Aged > 60 years
FRISBEE 2-year	Any type	0.34 (0.02–0.67)	0.23 (–0.13–0.60)
	Major osteoporotic	0.33 (–0.09–0.74)	0.30 (–0.22–0.83)
	Central	0.61 (0.16–1.06)	0.48 (–0.09–1.05)
FRISBEE 5-year	Any type	0.54 (0.13–0.95)	0.45 (–0.09–0.98)
	Major osteoporotic	0.43 (0.05–0.80)	0.39 (–0.10–0.87)
	Central	0.69 (0.31–1.08)	0.50 (–0.06–1.06)
Garvan 5-year	Any type	0.50 (0.19–0.82)	0.64 (0.23–1.05)
	Major osteoporotic	0.63 (0.22–1.04)	0.78 (0.27–1.29)
	Central	1.09 (0.54–1.65)	0.94 (0.30–1.58)
FRAX* 5-year	Major osteoporotic	0.67 (0.64–0.70)	0.76 (0.73–0.79)

*AOD*, anti-osteoporotic drugs; *Garvan*, Garvan nomograms; *FRAX*, Fracture Risk Assessment Tool. *Missing FRAX calculation for 7 patients (*n* = 510)

In the FRISBEE 5-year risk models, calibration plots also showed poor agreement between observed and predicted risks across the deciles of risks, and the deciles did not follow the unity line in the calibration plot for none of three types of fracture (Fig. 3D–F). Most of the deciles were clustered at the bottom left, indicating that there was an overestimation in risk of all fractures. The calculated calibration slopes were low for any fracture (0.54), MOF (0.43), and moderate for central fractures (0.69) (Table 3).

For the Garvan nomograms, the calibration plots for central fractures showed a calibration slope of 1.09 and 0.94, in the total cohort and in patients older than 60 years, and excellent agreement between observed and predicted risk of central fractures (Table 3, Fig. S2). For FRAX, the calibration plots for MOF showed a calibration slope of 0.67 in the total cohort (Table 3, Fig. S2).

## Discussion

In this study, we validated the 2-year and 5-year fracture prediction models of FRISBEE in a cohort of women aged 50–90 years with fractures. The FRISBEE 2-year and 5-year models did not possess an acceptable discriminatory capability and overestimated the fracture risk. However, the discriminative capability of the FRISBEE 5-year models in this cohort were comparable to Garvan and FRAX. Overall, Garvan showed best agreement between expected and observed risk for central fractures.

The FRISBEE risk models are based on data from the prospective FRISBEE cohort of 3560 women aged 60–85 years (mean age 70.1 ± 6.4 years) who were followed for 5 years. Of these, 881 (25%) women had experienced an index fracture. Of those, 130 (15%) sustained a second, imminent fracture during follow-up, and the mortality was 3% [10]. In comparison, all 517 women aged 50–90 years (mean age 65.5 ± 8.6 years) in the NoFRACT cohort were younger and had sustained an index fracture at inclusion, 53 (10%) sustained an imminent fracture during the first 2 years of follow-up, and the mortality was 5% after 5.6 years follow-up. Although all women in the NoFRACT cohort had an index fracture and higher expected risk of fractures and mortality, the proportion of imminent fractures was lower than in the FRISBEE cohort. One explanation could be lower age, but sensitivity analyses in the cohort above 60 years did not change the results. Another explanation could be the three times higher proportion of participants using AOD in the NoFRACT cohort than the FRISBEE cohort (56% vs. 19%) [6]. However, the incidence was not as low as in the individuals who were not prescribed AOD, who had a very low fracture risk at baseline. Unfortunately, the subgroups of patients using and not using AOD were too small to be studied separately. The proportion of participants who reported falls during the last year before inclusion was equal in the two cohorts (24% in NoFRACT and 25% in FRISBEE).

### FRISBEE 2-year prediction model

When we evaluated the calibration, all three FRISBEE 2-year models overestimated the risk of imminent fracture. All the AUC values were < 0.7, which indicate that the models did not possess an acceptable discriminatory capability [17]. Miscalibration is not uncommon in external validation of prediction tools [12], and one of the explanations can be differences in fracture risk between the cohorts. Norway has among the highest incidence rates of hip fractures in the world, higher than Belgium (420 vs. 370/1,000,000/year) [20]. Anyhow, this should more likely contribute to an underestimation than an overestimation of fractures, when using a Belgian model. The overestimation of fracture risk can also be influenced by the higher proportion of participants using AOD in the NoFRACT cohort than the FRISBEE cohort. In the development of the FRISBEE 5-year prediction models, no significant discrimination or calibration bias for treated patients was found, although there was a trend for a slightly weaker prediction in treated subjects (personal communication with Professor Jean-Jacques Body, Université Libre de Bruxelles, Brussels, Belgium). Treatment with AOD was not significant in their multivariable analyses and consequently was not considered a clinical risk factor in their models. When validating the 2-year risk models, we did not find any difference between the groups stratified for AOD, but the two groups were small and not comparable. It is important to keep in mind that the women who were using AOD had a high fracture risk at baseline, whereas those who was not treated with AOD had a low fracture risk. AOD treatment leads to fewer fractures than expected if untreated, with an overestimation of fracture risk as a result. We also noted that the expected 2-year risks of any type of fractures were 25% compared with 16% 5-year risk, which seems unlogic. However, antiresorptives have shown smaller impact on osteoporotic fractures after 2 years than after 5 years in randomized controlled trials [21–23], and this might be an explanation. As the mortality was only 5%, death did probably not explain the higher 2 years than 5-year risk of fractures.

### FRISBEE 5-year prediction model

The 5-year models also overestimated the fracture risk when applied on the total cohort. This is in line with the external validation of the 5-year FRISBEE prediction models in 9716 women aged 65–80 years from the Canadian Manitoba cohort [12]. They concluded that there was a need for recalibration before the use on a Canadian population and proposed that the explanations might be miscalibration, variation in fracture risk between countries, or differences between MOF/hip fracture ratio in the model than in the observed cohort.

### Garvan and FRAX

The only external validation of the 5-year model of FRISBEE until now concluded that further external validation on other international cohorts was warranted, and comparisons against other risk calculators could be of interest [12]. We therefore explored the performance of the well-known risk tools Garvan and FRAX, to compare with the FRISBEE validation results. For the Garvan 5-year risk model of any type, MOF, and central fractures, AUC was comparable to the results from the 5-year risk models of FRISBEE. Garvan performed best for central fractures. This might be because central sites of BMD measurements better predict centrally sited fractures [24]. For Garvan nomograms, the calibration plots for central fractures showed an excellent agreement between observed and predicted risk of central fractures. Anyhow, applied on the whole cohort, the FRISBEE models did not deviate too much from Garvan and FRAX.

### Strengths and limitations

A strength of this study is that the FRISBEE models were applied on a population with a high imminent fracture risk, where calculation of 2-year fracture risk is highly relevant to evaluate indication for AOD. To our knowledge, this is the first externally validation of the FRISBEE 2-year fracture risk model. Further, we validated the 5-year fracture risk model in the same population, showing the performance of these models over time. We also compared the validation results with Garvan and FRAX, which to our knowledge has not been published before. This study has also several limitations. The NoFRACT sub-study was not designed to validate the FRISBEE models. The moderate sample size of the NoFRACT cohort, including a low number of subsequent fracture events, raises question if the sample was too low for a proper validation. Another weakness was that, in the 2-year model predicting any type of fractures, two patients had an estimated imminent fracture risk > 100%. Both these patients had very low BMD (T-score <  − 4.0) and reported falls. In the calibration analyses, the risk was set to 99.9%. Another limitation could be that the fracture data was only registered from patients’ records. Potentially, a chart review as the sole source of data on new fractures may render a too low number, especially for ribs and vertebral fractures. However, in Norway, patients are assigned to one hospital according to home address, so even if they got their primary fracture treatment while travelling, further treatment is almost always undertaken at their assigned hospital. Further, in NoFRACT, most of the women with index hip or vertebral fracture were directly treated with zoledronic acid or denosumab during the hospital stay for the index fracture and were never referred to DXA examination and included in the sub-study. This could have led to a healthy selection bias and explain the overestimation of the fracture risk in the FRISBEE model, as well as in the Garvan and FRAX models.

To conclude, in this cohort of Norwegian women with fractures, the FRISBEE 2-year and 5-year fracture prediction models did not have an acceptable discriminatory capability and overestimated the fracture risk. However, the discriminative capability of the 5-year models in this cohort was comparable to Garvan and FRAX. Overall, Garvan showed the best agreement between expected and observed risk for central fractures. Applied on the whole cohort, the different models overestimated the fracture risk, which suggests that there is a need for recalibration before using the models in a Norwegian FLS cohort. A tool for estimating imminent fracture risk is warranted, and the FRISBEE prediction models should also be validated in other cohorts.

## Supplementary Information

Below is the link to the electronic supplementary material.

ESM 1 (PNG 94.2 KB)

ESM 2 (PNG 102 KB)

Supplementary file1 (TIF 4799 KB)

Supplementary file2 (TIF 4799 KB)
