# Supplementary figures and images for: External validation of FRISBEE 2-year and 5-year fracture prediction models in a fracture liaison service cohort
Source: Arch Osteoporos. 2025 Aug 2;20(1):107. doi: 10.1007/s11657-025-01516-5 (PMC12316711; doi:10.1007/s11657-025-01516-5)

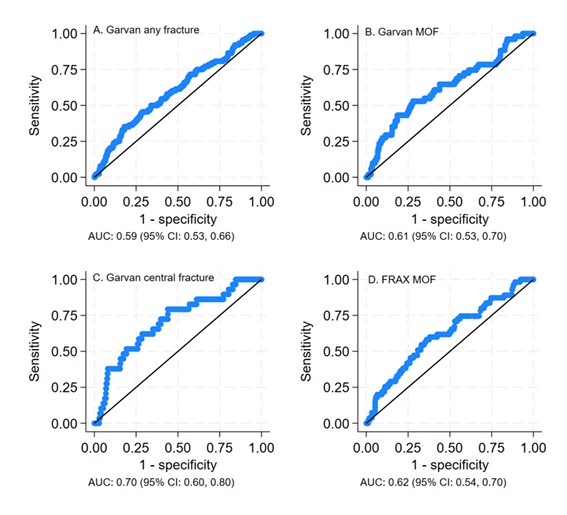

Supplement: Supplementary file 1 — (PNG 94.2 KB) [file 11657_2025_1516_Fig4_ESM.png]

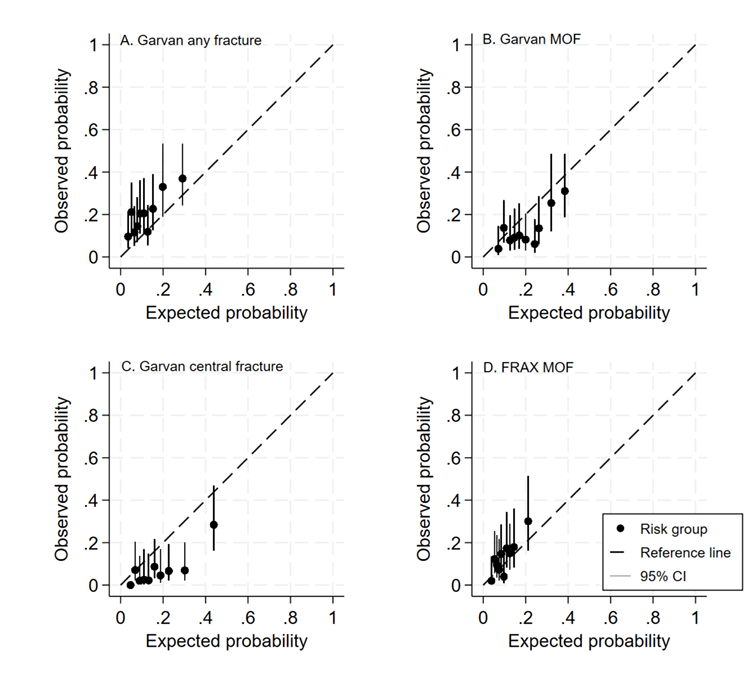

Supplement: Supplementary file 2 — (PNG 102 KB) [file 11657_2025_1516_Fig5_ESM.png]

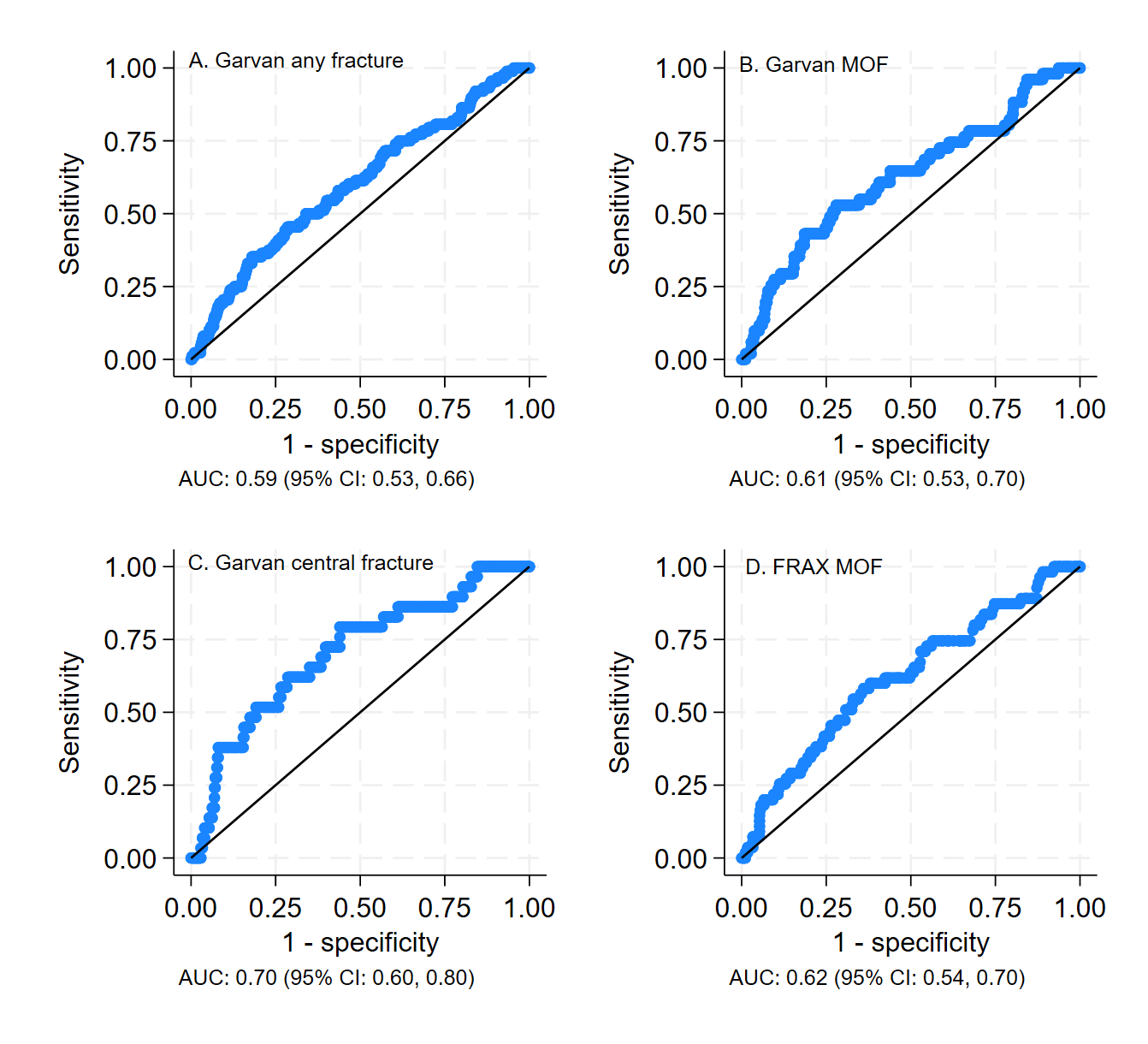

Supplement: Supplementary file 3 — Supplementary file1 (TIF 4799 KB) [file 11657_2025_1516_MOESM1_ESM.tif]

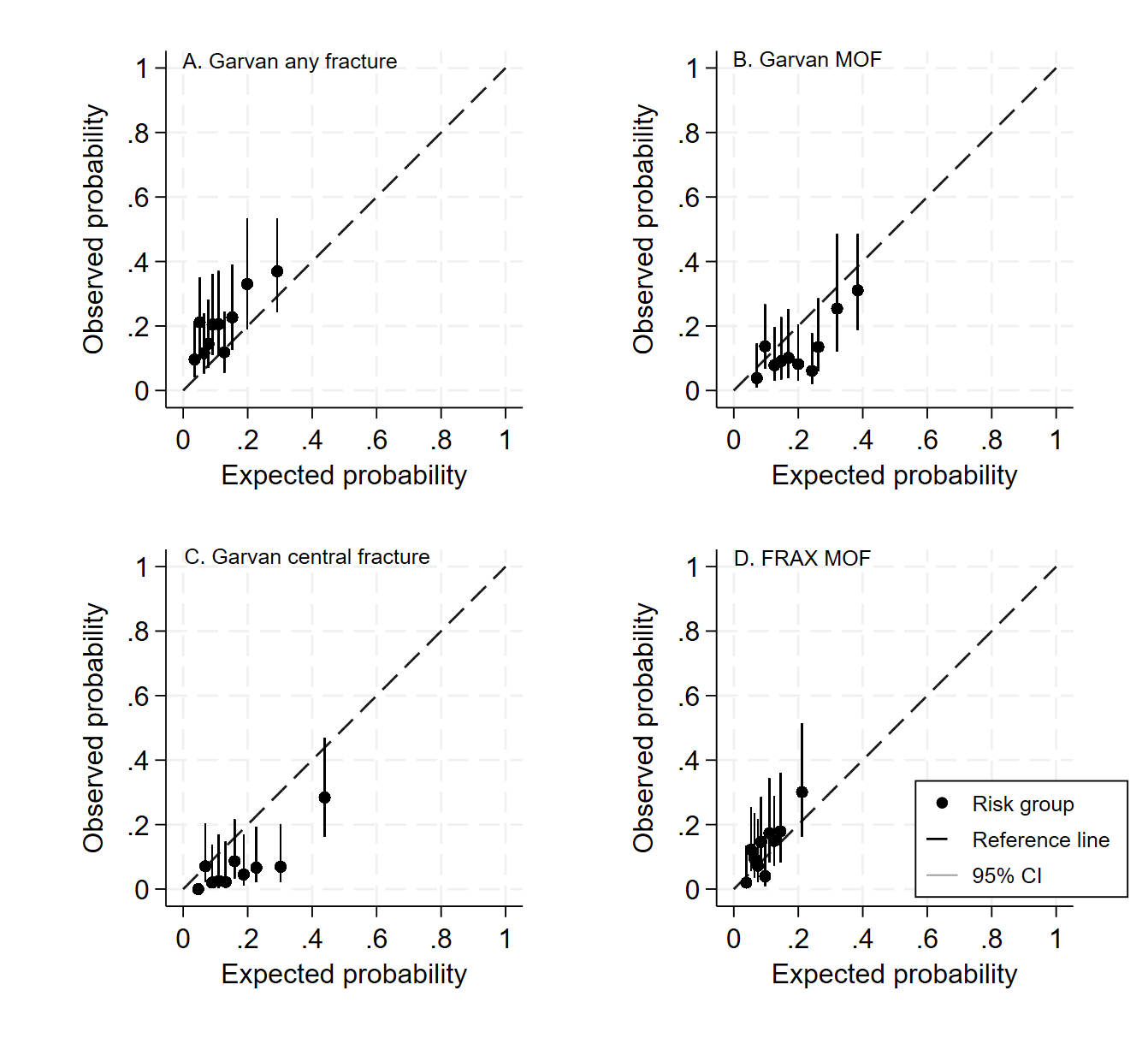

Supplement: Supplementary file 4 — Supplementary file2 (TIF 4799 KB) [file 11657_2025_1516_MOESM2_ESM.tif]
